# A naturalistic virtual reality task reveals difficulties in time-based prospective memory and strategic time-monitoring in children with ADHD

**DOI:** 10.1038/s41598-025-08944-w

**Published:** 2025-07-09

**Authors:** Erik Seesjärvi, Sascha Zuber, Emilie Joly-Burra, Matthias Kliegel, Juha Salmi

**Affiliations:** 1 Department of Psychology and Logopedics, University of Helsinki, Helsinki, Finland; 2 Child Neurology, University of Helsinki and Helsinki University Hospital, Helsinki, Finland; 3 Center for the Interdisciplinary Study of Gerontology and Vulnerability, University of Geneva, Geneva, Switzerland; 4 Swiss National Centre of Competence in Research LIVES – Overcoming vulnerability: Life course perspectives, Universities of Lausanne and of Geneva, Geneva, Switzerland; 5 Faculty of Psychology and Educational Sciences, University of Geneva, Geneva, Switzerland; 6 Unit of Psychology, Faculty of Education and Psychology, Oulu University, Oulu, Finland; 7 Department of Neuroscience and Biomedical Engineering, Aalto University, Espoo, Finland

**Keywords:** ADHD, Virtual reality, Ecological validity, Time-based prospective memory, Future intentions, Human behaviour, Paediatrics

## Abstract

Time-based prospective memory (TBPM), which supports executing everyday actions at appropriate times, is a key cognitive skill generally weaker in individuals with attention-deficit/hyperactivity disorder (ADHD). To better understand the mechanisms of TBPM, we used a naturalistic virtual reality task called Executive Performance in Everyday LIving (EPELI) to study TBPM in 71 children with ADHD and 71 typically developing peers aged 9–13 years in an ecologically relevant context. Our results revealed that children with ADHD showed lower performance on TBPM tasks not because of how frequently they monitored the time overall, but because they did so less strategically. More specifically, the degree of strategic time monitoring accounted for 22.1% of variance in TBPM performance and fully mediated the effect of ADHD. Together, the absolute frequency of clock-checking, the degree of strategic time monitoring, and ADHD status explained 53.9% of the variance in TBPM performance. As monitoring strategies can be trained, the present findings may have important implications for targeted support to help individuals with ADHD manage their schedules.

## Introduction

Prospective memory (PM), the ability to perform previously planned tasks in the future^1^, is essential for independent daily functioning^2–5^. In childhood, PM can be regarded as one of the cornerstones of cognitive development^6^. Already during primary school, children are expected to remember and execute intended tasks on their own^7^. For example, children need to remember to leave for school or their hobby on time and monitor how much time they have to prepare for the next task. Several factors have been recognized that contribute to typical and atypical development of PM^8–11^. These include, for instance, retrospective memory, executive functions (planning, working memory, inhibitory control, monitoring), attention, metamemory, and motivation. A variety of PM tasks have been developed to study the development of PM^11^. Studies show that related skills emerge early (before the age of three years) but continue to develop throughout childhood^11^. Atypical developmental trajectories in children’s PM can adversely affect their daily functioning across key life domains, such as academic performance and social relationships^8^.

Attention-deficit/hyperactivity disorder (ADHD), which affects an estimated 5 to 11 percent of individuals worldwide^12^, is one of the most common neurodevelopmental conditions linked to reduced PM performance^13,14^. Because of the importance of PM for carrying out activities of daily-life, clarifying how ADHD affects PM has considerable implications for a large group of children, their families, and health care system. Previous research has shown that children with ADHD may experience greater challenges than their peers in time-based PM (TBPM) than in event-based PM^13,14^. TBPM allows intended tasks to be performed either at a specific time in the future (e.g., at 10 am) or after a certain time has elapsed (e.g., after 10 min), whereas event-based PM triggers our memory when a specific event occurs (e.g., remembering to hand over a birthday invitation when running into a friend). Compared to event-based tasks, time-based tasks rely more strongly on self-initiated processes^15,16^. More specifically, to detect the right moment to perform a PM action, time-based tasks require participants to spontaneously remember to monitor the passage of time in the absence of clear external cues. This may be particularly difficult for children with ADHD, as symptom definitions highlight difficulties with sustained attention, time management, and forgetfulness in daily activities^17,18^. Time monitoring has often been operationalized as the number of clock checks (absolute clock-checks) before the target time has elapsed (e.g., ^19^), but other studies have also examined how clock checks are distributed across time leading up to the target moment, which has become a standard approach in the field (for a review, see ^20^).

Most studies on TBPM performance show small-to-large effect sizes favoring typically developing children over those with ADHD^21–24^. The only exception was a study by Yang et al.^25^, which found no group differences likely because most of their ADHD participants were taking stimulant medication, whereas children in the other studies were tested unmedicated. Hence, the evidence of TBPM difficulties in children with ADHD is solid, although the underlying mechanisms remain poorly understood.

When examining the overall frequency of time monitoring, previous studies have not found group differences in how frequently typically developing children versus children with ADHD check the clock during TBPM tasks. Studying temporal distribution of clock checks by dividing the time before the target time into four intervals of equal length, both Zinke et al.^24^ and Mioni et al.^22^ found that the frequency of clock checks increased as the target time approached in both groups. Mioni et al.^22^ also found that children with ADHD checked the time less often than typically developing children in the last (fourth) interval before the target time, while the opposite was true for the first interval after each target time. This was interpreted to indicate less strategic time monitoring in children with ADHD, as checking the time may take cognitive resources from other tasks at hand but becomes more critical for successful TBPM performance as the target time approaches. However, this was not observed by Zinke et al.^24^, suggesting that potential difficulties in strategic time monitoring in children with ADHD warrant further investigation.

Several studies have also investigated associations between TBPM performance and time monitoring^21,22,24^. In their first experiment, Kerns and Price^21^ found that monitoring time more frequently predicted better TBPM performance in typically developing children, but not in children with ADHD. In their second experiment, however, this was not replicated as such correlation was not observed in either group. Zinke et al.^24^ reported that the number of clock checks in the last interval before the PM target time predicted better TBPM performance both in children with ADHD and typically developing children. The authors found that controlling for the total number of clock checks reduced the group difference in TBPM performance by a small amount. Importantly, however, when controlling specifically for the number of clock checks during the last interval, the group difference was no longer significant. Furthermore, Mioni et al.^22^ found that for children with ADHD, only the number of clock checks in the last (fourth) interval before the target time correlated with TBPM performance, whereas for typically developing children, the number of clock checks in the second and third intervals before target time also showed significant correlations. Taken together, research suggest that clock checks right before target time, indicating strategic time monitoring, may be especially important for successful TBPM performance, and may help explain differences in TBPM among unmedicated children with ADHD and typically developing children.

While the above-mentioned studies have established several key findings related to TBPM of children with ADHD, they have some important limitations. First, while the TBPM challenges in ADHD are relatively well documented, the behavioral pathways underlying these deficits are much less clear. This particularly concerns the role of time monitoring as a key mechanism underlying group and individual differences in TBPM. Specifically, focusing only on the frequency of clock checks in the last interval before TBPM target time, without considering the overall number of clock checks, may not be the optimal approach for assessing how strategic an individual’s time monitoring is^26^. This is because participants with many clock checks in the fourth and last interval may simply also check the time more often in earlier intervals (rather than doing so specifically in the last interval ), which can be considered as suboptimal use of cognitive resources^26^. Furthermore, previous studies have not examined the effects of both frequency of time monitoring and the level of strategic time monitoring within the same analysis. In a recent study on healthy adults aged between 19 and 86 years, Joly-Burra et al.^26^ suggested that to properly distinguish between these two aspects of time monitoring, the level of strategic time monitoring should be operationalized by examining the ratio of clock checks within the last interval before PM target time to overall number of clock checks. Using this new indicator for the level of strategic time monitoring (called “relative clock-checking”), the authors found that the absolute frequency of clock-checking and the level of strategic clock-checking together fully accounted for the negative age-gradient in TBPM and explained 53.6% of the variance in TBPM performance. Both aspects of time monitoring were needed to fully mediate the age effect on TBPM, but strategic clock-checking was a stronger predictor of TBPM performance than the total frequency of clock-checking. The authors proposed that the absolute frequency of clock-checking reflects the level of resources that participants can devote to monitoring the time, while relative clock-checking reflects how they make use of these resources and deploy them strategically over time (see also^27,28^). Wang et al.^29^ examined relative clock-checking (time monitoring) in typically developing children aged 7 to 11 years and found that older children were better able to allocate their attentional resources and use time monitoring strategies to enhance their TBPM performance. In the present study, we perform a similar analysis to examine whether TBPM deficits related to children’s ADHD are mediated by differences in the frequency of time monitoring, the degree of strategic time monitoring, or both.

The role of task type has been widely acknowledged in developmental studies of PM^11,30^. The type of paradigms used can be considered as an important limitation in the aforementioned TBPM studies involving children with ADHD. Kerns and Price^21^ and Talbot and Kerns^23^ employed a 2-dimensional video-game like task where children can maneuver their vehicle to avoid collisions with other vehicles, check the gas level (i.e., time monitoring), and refuel when gas is low (i.e., PM task), while the speed of the game is constant throughout the task. Zinke et al.^24^, in turn, used a 1-back task where the children were asked to give dichotomic (i.e., yes/no) answers to target and non-target stimuli delivered at constant inter-stimulus intervals; remember to press a PM target button every two minutes (i.e., TBPM task); and monitor time when needed by pressing another button. In Mioni et al.^22^, children watched a cartoon while having the possibility to check the time by pressing a green button and being instructed to press a red button every two minutes. To summarize, none of these paradigms involved children engaging in free-paced activities in a stimulus-rich environment that allows for varied and spontaneous behavioral responses. This is an important limitation as difficulties in staying organized in open-ended situations is one of the key challenges in ADHD. In PM research, there is a well-known paradox that younger adults tend to outperform older adults in typical computerized laboratory paradigms, while older adults perform at least comparably well to, and sometimes even better than, younger adults in more naturalistic tasks performed in everyday contexts^31–34^. This highlights the importance of studying PM also in conditions that more closely resemble everyday life.

To address these and related challenges of ecological relevance we recently developed a virtual reality (VR) task called “Executive Performance in Everyday LIving” (EPELI) that can be used to quantify goal-directed behavior in naturalistic but controlled settings^35,36^. EPELI allows children to freely explore a stimulus-rich virtual apartment resembling a typical home and to interact with its objects at their own pace in 13 different scenarios. At the beginning of each scenario, children hear instructions for a set of tasks they should complete (e.g., morning or evening activities or preparing to go to school or a hobby). Children then carry out these tasks while navigating the environment that includes many task-irrelevant distractors, like ambient sounds and a visible, audible fly. Compared to the more traditional laboratory PM paradigms mentioned above, EPELI shares greater resemblance to everyday life, making it more ecologically relevant in terms of verisimilitude^35,37^. In a previous study, children with ADHD showed lower attentional-executive efficacy and executed more impulsive actions and hyperactive movements in EPELI compared to their typically developing peers^35^ (see also^38^). Importantly, we also found that the fluctuations of attention associated with ADHD, as reflected in response latencies, may manifest quite differently in a more traditional, externally-paced laboratory task (i.e., a continuous performance task, CPT) compared to EPELI, where participants are free to proceed at their own will and pace^39^. For example, in EPELI, children with ADHD showed shorter response latencies than their typically developing peers, whereas in the CPT, the reverse pattern was observed. Group differences in ex-Gaussian parameter *τ* (tau), which reflects occasional sluggish responses, were more pronounced for the ADHD group in the CPT. In EPELI, however, these differences depended on task relevance of each action: children with ADHD showed smaller tau values on task-relevant actions but larger tau values on task-irrelevant ones. Thus, ADHD-related difficulties in TBPM and related time-monitoring behavior may also differ during externally and internally paced situations.

To better understand *whether* and *why* children with ADHD display more difficulties with TBPM, the present study employed a naturalistic, free-paced VR task depicting several real-life scenarios. More precisely, we set out to examine, if, in the context of EPELI, 1) children with ADHD differ from typically developing peers in TBPM performance, 2) the two groups display differences in the frequency of time monitoring or the degree of strategic time monitoring, and 3) potential differences in time monitoring mediate (i.e., account for) possible group differences in TBPM performance. Based on previous literature, we hypothesized that children with ADHD would show lower TBPM performance (see^21–25^) and show an equal frequency of time monitoring compared to typically developing peers (see ^22,24^). Based on Mioni et al.^22^, we predicted that children with ADHD would show less strategic time monitoring (cf.^24^). Furthermore, the findings from Joly-Burra et al.^26^ led us to anticipate that group differences (ADHD vs. typically developing) in TBPM performance are mediated by time monitoring.

## Methods

### Participants

The data was combined from two previous studies^35,38^. For the participants in the ADHD group, the inclusion criteria were (a) native language Finnish, b) age of 9 to 12 years when recruited, and (c) an ADHD diagnosis (F90 in ICD-10^18^), while the exclusion criteria were (a) any diseases of the nervous system (G00–G99) and (b) any other mental or behavioral disorders (F00–F99) except for ADHD. As an exception to the exclusion criteria (b), Emotional disorder with onset specific to childhood (F93) and Unspecified behavioral and emotional disorder (F98) were allowed as secondary diagnoses given their common co-occurrence with ADHD. In the ADHD group, 60 children had a medicine prescribed for their ADHD symptoms^35,38^, but they took part in the study unmedicated (24 h washout period). For the participants in the typically developing control group, the same criteria applied, except all mental or behavioral disorders (F00–G99) and the need for special support in school (e.g., a placement in a small group) were considered exclusion criteria. The combined sample first included 71 children with ADHD and 81 typically developing controls (see Supplementary Table 1). After propensity matching the groups regarding age and sex, the final sample comprised 71 participants with ADHD and 71 controls (see Table 2). The study was conducted in accordance with the Declaration of Helsinki and performed under approval from the Ethics Committee of the Helsinki University Hospital (HUS/1589/2019). Informed consent was received from all children and their legal guardians. All families were compensated with two movie tickets for participating in the study.

### Measures and procedure

#### EPELI task

**Fig. 1:**
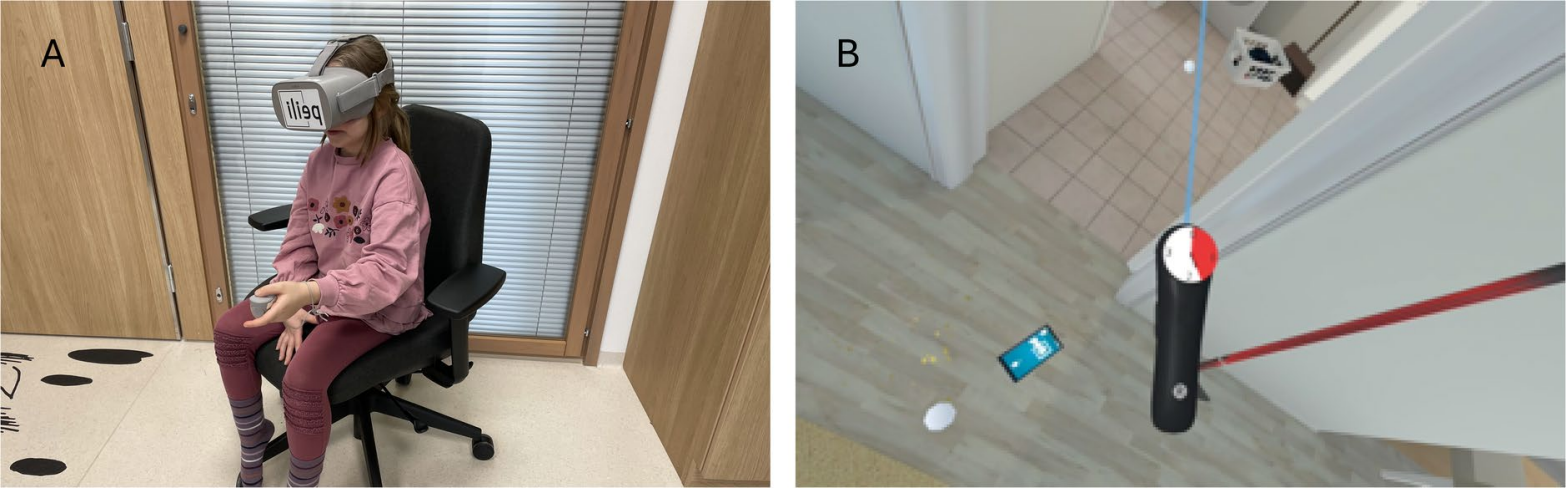
(**A**) a child playing EPELI, (**B**) a screenshot from EPELI showing the controller with the clock face.

EPELI is a VR-based task where participants perform everyday chores in a virtual home environment (see https://aalto.cloud.panopto.eu/Panopto/Pages/Embed.aspx?id=3eb4836f-1238-4f27-853a-ad3700745b31;^35,36^). In this study, all children performed EPELI using a head-mounted display, either Oculus GO (participants from Seesjärvi et al.^35^) or Pico Neo 2 Eye (from Merzon et al.^38^). To ensure participant safety, children performed EPELI in a sitting position on a revolving chair (Fig. 1A). They could look around by rotating their head and interact with objects using a hand controller that was also seen in the virtual environment. In the environment, the user was able to interact with or pick up objects targeted by a ray from the hand controller with the click of a button. If an object had been picked up, it could be dropped by pointing at a desired location with the hand controller and clicking the button again. The time could be checked by raising or tilting the hand controller slightly and looking at it. This gesture would reveal a clock face, divided into four quadrants indicated by numbers 0, 1 (corresponding to 15 s), 2 (corresponding to 30 s), and 3 (corresponding to 45 s; see Fig. 1B). When the clock reached 0 again, the clock-face was fully red, indicating that 60 s had passed.

The version of EPELI used in the study included 13 short scenarios with different themes, each containing one TBPM task (Table 1). For example, Scenario A presented a situation in which the child has just woken up and must complete their morning chores (for a complete example scenario, see the Supplementary Material). Each scenario started with an instruction phase lasting 22–47 s, where a cartoon dragon orally explained the tasks-to-be-completed, including the TBPM task. In the subsequent execution phase, the children were told to perform the tasks “as instructed before” without any explicit reminders. While completing the tasks, they were free to navigate the apartment and interact with various objects, of which many were irrelevant to the tasks at hand. Each scenario had four to seven tasks. For each scenario, one of these tasks was a TBPM task and the others were considered as the ongoing task, into which the TBPM task was embedded. Depending on the scenario, the TBPM task was to be executed at 1 o’clock (15 s in real time), 2 o’clock (30 s in real time), or 3 o’clock (45 s in real time). The time on the clock started running at the beginning of each execution phase, except in two scenarios (B and L), where it began only after the child had completed another task related to the TBPM task. Each execution phase ended when the given tasks had been completed, or after 90 s. Overall, the children in the study completed EPELI within a 23 to 36 min range, depending on how quickly they performed the free-paced demo session; how successful they were performing all the given tasks; and whether they needed to take short breaks between the scenarios. Half of the participants performed scenarios in alphabetical order (A to M) and half of the participants performed the scenarios in reverse order.

**Table 1:** EPELI scenarios.

Scenario	Tasks	TBPM task	TBPM target time
A. Waking up	4	Put the porridge on the stove	1
B. Cooking carrot soup	7	Turn off the stove	2
C. Leaving for school	5	Call mom	3
D. Play time 1	6	Call your friend	1
E. Returning from school	5	Turn on the oven	3
F. Day off	5	Call grandmom	2
G. Leaving for soccer practice	6	Turn on the oven	2
H. Play time 2	6	Call grandmom	2
I. Going to bed	6	Turn off the radio	3
J. Play time 3	6	Watch the tv	1
K. A friend is coming over	5	Turn off the bathtub tap	3
L. Returning from soccer practice	5	Take a bath	3
M. Cleaning day	4	Call your sister	2

The number of tasks includes the TBPM task. Target time 1 corresponds to 15 s in real time, 2 to 30 s in real time, and 3–45 s in real time.

Four measures were analyzed from EPELI. *TBPM performance* was the percentage of TBPM tasks that were executed within + / − 10 s of their target time^36^. *Ongoing task performance* was the percentage of all correctly performed tasks in EPELI^35^ but excluding TBPM tasks. The time-monitoring measures, that is, absolute clock-checks and relative clock-checks, were adapted from Joly-Burra et al.^26,27^. *Absolute clock-checks* was the total number of clock checks from the beginning of each scenario until the TBPM target time. To calculate *relative clock-checks*, we first divided the time between when the clock started running and TBPM target time in three intervals of equal length (T1–T3). Depending on the target time (15, 30, or 45 s), each interval was 5, 10 or 15 s long, respectively. Then, within each scenario, the number of clock checks during the last interval (T3) was divided by the total number of clock checks in all intervals. The scenarios with no clock checks were regarded as missing values.

In addition to EPELI, the children performed two subtests from the Wechsler’s Intelligence Test for Children—fourth edition (WISC-IV): Similarities and Matrix Reasoning^40^. From these subtests, standardized scores were used as background information. For further background information, the parents were asked for their average income and to fill in the ADHD Rating Scale-IV (ADHD-RS)^41^.

The measurements were conducted in dedicated rooms in schools or at university facilities (Aalto Behavioral Laboratory, Aalto University; Åbo Akademi University). For further details, see the previous studies^35,38^.

### Statistical analyses

Statistical analyses and data visualization were performed in R version 4.0.3^42^ using additional packages data.table^43^, ggplot2^44^, MatchIt^45^ and lavaan^46^.

Before the analyses, the groups were matched regarding age and sex to eliminate the possible confounding effects of these background factors. This was done by calculating a propensity score for each participant with the MatchIt package^45^ and excluding participants with most deviating scores from the control group until no differences in these background variables were found. This led to the exclusion of 10 participants from the control group. After this, outlier analysis was performed for the key PM variables of interest (TBPM performance, absolute clock-checking and relative clock-checking). As one participant in the ADHD group and one in the control group deviated substantially from their respective group means (i.e., > 3.5 SDs), we employed maximum likelihood estimation with robust standard errors (MLR) in the structural equation modeling, as recommended by Cousineau and Chartier^47^ for handling non-normally distributed data. To assess the robustness of our findings, we also reanalyzed the data after excluding these two participants and using standard maximum likelihood estimation without MLR. This reanalysis yielded highly similar results with no differences in the interpretation of findings. As such, we report only the results obtained without excluding participants with outlying scores.

Linear relationships between the four PM-related measures were indicated by Pearson’s correlation coefficients and interpreted, when statistically significant, as weak (0.10–0.29), moderate (0.30–0.49), and strong ($\ge \;0.{5}0$)^48^.

To examine if ADHD affects time monitoring or TBPM performance and if time monitoring mediates a possible group (ADHD vs. control) effect on TBPM performance, several structural equation models were estimated with *lavaan* package^46^. In Model 0, TBPM was regressed on group only. In Model 1, we fitted a mediation model in which both absolute and relative clock-checking mediated the group effect on TBPM. Thus, absolute clock-checking, relative clock-checking and TBPM were regressed on group, and TBPM was further regressed on absolute and relative clock-checking. The indirect effects of group on TBPM through absolute and relative clock-checking were estimated and are reported as indexes of mediation^49^. Total effect of group on TBPM was estimated by summing the direct and indirect group effects (through absolute and relative clock-checking). To test the difference between the two coefficients of two clock-checking measures on TPBM, we fit another alternative model (Model 1b), in which the coefficients were constrained to be equal. Model 2 was identical to Model 1, but all effects related to relative clock-checking were removed. In turn, Model 3 was equal to Model 1, but without the effects related to absolute clock-checking. All models provided a good fit to the data (CFI = 1.000, RMSEA < 0.001, SRMR < 0.001). To evaluate how much absolute and relative clock-checking each independently explained TBPM performance, two additional models with each clock-checking measure as the sole predictor were fitted. Of these models, only the variance explained is reported.

## Results

### Background characteristics

Background characteristics of the sample can be found in Table 2. After propensity matching, the groups did not differ in terms of sex or age. As expected, children in the ADHD group had more parent-reported problems in ADHD-RS. They also came from lower-income families and scored lower on both verbal and non-verbal reasoning tasks.

**Table 2:** Background variables.

Variable		Group		Test statistics	*p*
		ADHD (n = 71)	Control (n = 71)		
Age (SD)		10.4 (1.1)	10.7 (1.0)	*t*(140) = − 1.553	.123
Sex	Boy	58	49	Fisher’s exact test	.119
	Girl	13	22		
Average parental income before tax (%)	Less than 1,500 €/m	2 (2.8)	0 (0.0)	W = 1678^a^	< .001***
	1,500–2,200 €/m	4 (5.6)	2 (2.8)		
	2,200–3,000 €/m	27 (38.0)	10 (14.1)		
	3,000–4,000 €/m	15 (22.5)	20 (28.2)		
	over 4 000 €/m	22 (31.0)	39 (54.9)		
Verbal reasoning (SD)		10.4 (2.7)	11.5 (2.5)	*t*(143) = − 2.850	.009**
Non-verbal reasoning (SD)		9.5 (3.1)	10.6 (3.2)	*t*(143) = − 2.230	.040*
ADHD-RS (SD)		32.0 (8.9)	6.9 (6.3)	*t*(143) = 19.686	< .001***

SD, standard deviation; Verbal reasoning, WISC-IV Similarities standard score; Non-verbal reasoning, WISC-IV Matrix Reasoning standard score; ADHD-RS, ADHD rating scale-IV.

^a^ = Wilcoxon rank sum test with continuity correction.

* = *p* < .05, ** = *p* < .01, *** = *p* < .001.

### Correlations between TBPM performance, time monitoring, and ongoing task performance

Descriptive statistics for the PM measures and ongoing task performance, along with their correlations, are presented in Table 3. TBPM performance was strongly correlated with the ongoing task performance, indicating that children with better TBPM performance were also more successful in completing the other instructed tasks. TBPM performance was moderately correlated with both absolute and relative clock-checking, but the two time monitoring measures did not correlate with each other, suggesting that there was no relationship between the frequency of time monitoring and the level of strategic time monitoring. Ongoing task performance correlated weakly with absolute clock-checking and moderately with relative clock-checking.

**Table 3:** Descriptive statistics for the PM measures, ongoing task performance, and their correlations.

	Group								Correlations		
	ADHD (n = 71)				Control (n = 71)				1	2	3
	Mean	SD	Skew	Kurt	Mean	SD	Skew	Kurt			
1. TBPM performance %	32.7	17.4	0.458	0.241	39.3	18.8	0.050	− 0.542			
2. OT performance %	73.6	12.6	− 0.577	0.567	80.9	9.1	− 0.777	0.580	.61***		
3. Absolute clock-checking	23.6	12.4	2.159	9.067	21.2	12.5	1.358	2.853	.49***	.23**	
4. Relative clock-checking	17.6	12.2	1.264	2.079	26.7	14.1	0.376	0.064	.47***	.33***	− .11

SD, standard deviation; OT, ongoing task, Skew, skewness; Kurt, kurtosis.

* = *p* < .05, ** = *p* < .01, *** = *p* < .001 (false discovery rate correction).

### Effects of group and time monitoring

**Fig. 2:**
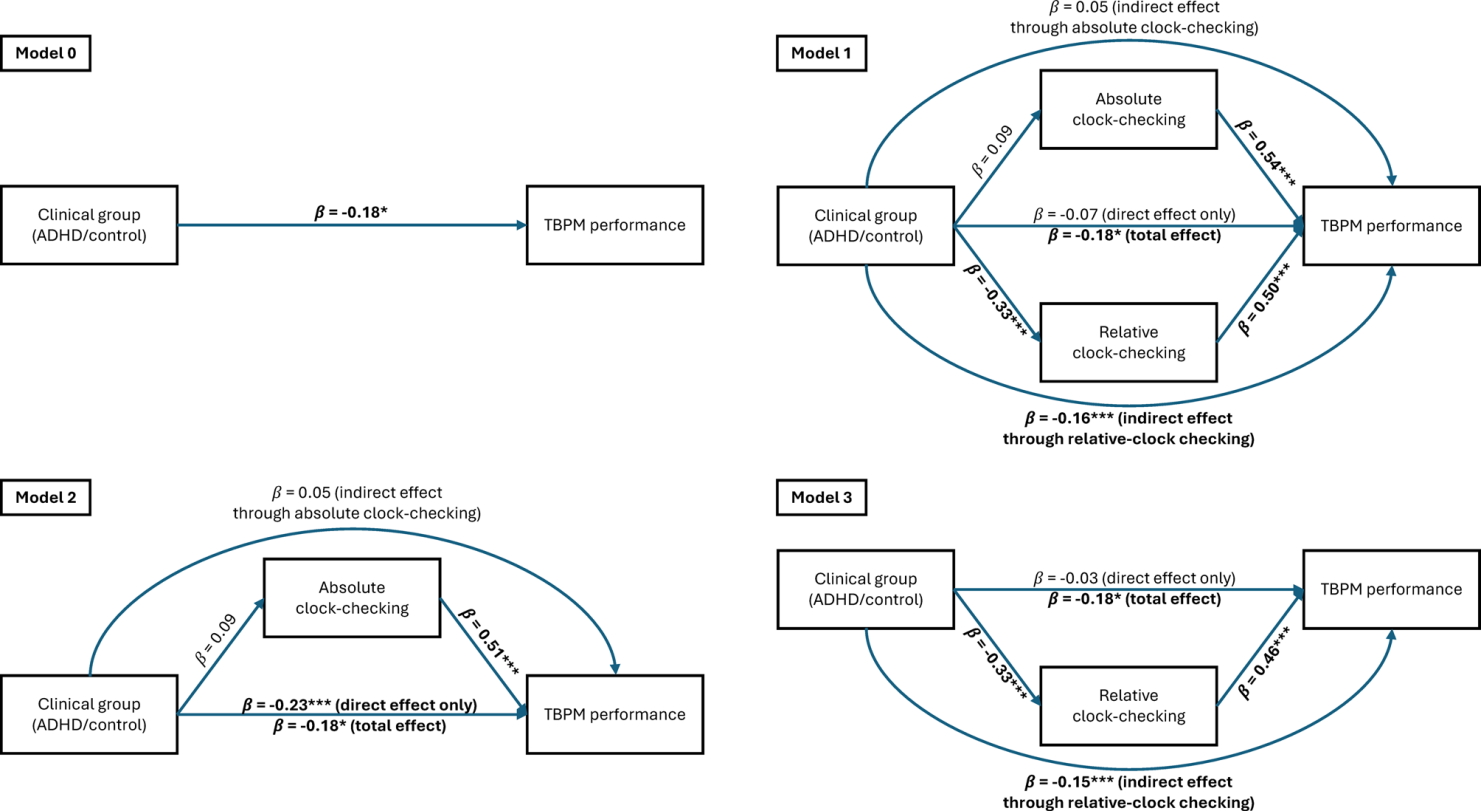
Illustration of Models 0, 1, 2 and 3. Upper curved arrows depict the indirect group effect on TBPM performance through absolute clock-checking. Lower curved arrows depict the indirect group effect on TBPM performance through relative clock-checking. Values are standardized estimates. Statistically significant effects are shown in bold. * = *p* < .05, ** = *p* < .01, *** = *p* < .001.

Effect estimates for Models 0, 1, 2 and 3 are presented in Table 4 and a visual representation in Fig. 2.

**Table 4:** Estimates for models 0, 1, 2 and 3.

Effect	Estimate	SE	*Z*	*p*	95% CI	*β*
MODEL 0						
**Group − > TBPM performance**	** − 6.609**	**3.019**	** − 2.189**	**.029**	**[− 12.526, − 0.692]**	**− 0.18**
MODEL 1						
Group − > TBPM performance	− 2.418	2.339	− 1.034	.301	[− 7.002, 2.167]	− 0.07
Group − > Absolute CC	2.338	2.077	1.126	.260	[− 1.733, 6.409]	0.09
**Group − > Relative CC**	**− 9.078**	**2.200**	**− 4.127**	**< .001**	**[− 13.390, − 4.766]**	**− 0.33**
**Absolute CC − > TBPM performance**	**0.809**	**0.070**	**11.556**	** < .001**	**[0.672, 0.946]**	**0.54**
**Relative CC − > TBPM performance**	**0.670**	**0.088**	**7.574**	**< .001**	**[0.497, 0.843]**	**0.50**
Group − > Absolute CC − > TBPM performance	1.891	1.666	1.136	.256	[− 1.373, 5.156]	0.05
**Group − > Relative CC − > TBPM performance**	**− 6.083**	**1.678**	**− 3.626**	**< .001**	**[− 9.371, − 2.795]**	**− 0.16**
**Group total**	**− 6.609**	**3.019**	**− 2.189**	**.029**	**[− 12.526, − 0.692]**	** − 0.18**
MODEL 2						
**Group − > TBPM performance**	**− 8.362**	**2.587**	**− 3.233**	**.001**	**[− 13.432, − 3.292]**	**− 0.23**
Group − > Absolute CC	2.338	2.077	1.126	.260	[− 1.733, 6.409]	0.09
**Absolute CC − > TBPM performance**	**0.750**	**0.069**	**10.905**	**< .001**	**[0.615, 0.885]**	**0.51**
Group − > Absolute CC − > TBPM performance	1.753	1.555	1.128	.260	[− 1.295, 4.801]	0.05
**Group total**	**− 6.609**	**3.019**	**− 2.189**	**.029**	**[− 12.526, − 0.692]**	**− 0.18**
MODEL 3						
Group − > TBPM performance	− 1.103	2.924	− 0.377	.706	[− 6.834, 4.628]	− 0.03
**Relative CC − > TBPM performance**	**0.606**	**0.115**	**5.257**	**< .001**	**[0.380, 0.833]**	**0.46**
**Group − > Relative CC**	**− 9.078**	**2.200**	**− 4.127**	**< .001**	**[− 13.390, − 4.766]**	**− 0.33**
**Group − > Relative CC − > TBPM performance**	**− 5.506**	**1.617**	**− 3.404**	**.001**	**[− 8.675, − 2.336]**	**− 0.15**
**Group total**	**− 6.609**	**3.019**	**− 2.189**	**.029**	**[− 12.526, − 0.692]**	**− 0.18**

SE = standard error; CI = confidence interval; β = standardized beta coefficient.

Statistically significant effects are shown in bold.

Group, 0 = control and 1 = ADHD; CC = clock-checking.

#### Effects of group on TBPM performance

In Model 0, there was a small effect of group on TBPM performance with the ADHD group displaying lower performance than the control group (*β* = − 0.18). In Model 1, the same effect was found as indicated by the total effect of group on TBPM performance (*β* = − 0.18), but when accounting for the significant indirect effect of group through relative clock-checking on TBPM performance, the direct effect of group was no longer significant. Therefore, relative clock-checking fully mediated the effect of group on TBPM performance. There was no indirect group effect on TBPM performance through absolute clock-checking. In Model 2, which included only absolute but not relative clock-checking, group again predicted TBPM performance as indicated by its direct effect (*β* = − 0.23) and total effect (*β* =  − 0.18). There was no effect of group on absolute clock-checking, hence no indirect effect of group on TBPM through absolute clock-checking was found. In Model 3, which comprised relative clock-checking as the sole time monitoring indicator, there was an indirect effect of group on TBPM performance through relative clock-checking (*β* =  − 0.15) and total effect of group (*β* = − 0.18), but the direct effect was no longer significant, as relative clock-checking fully mediated the effect of group on TBPM performance. To assess if the superior TBPM performance of the control group was at the expense of the other tasks-to-be-completed, the effect of group on ongoing task performance was tested separately. This analysis revealed a medium-sized effect of group on ongoing task performance with the ADHD group performing lower than the control group also here (*t*(140) = − 3.991, *p* < 0.001, *d* =  − 0.67).

#### Effects of group on time monitoring

In Model 1, there was a moderate effect of group on relative clock-checking with the ADHD group exhibiting lower values (i.e., less strategic time monitoring; *β* = − 0.33), but not on absolute clock-checking (i.e., the total number of clock checks; *β* = 0.06, n.s.). These findings were replicated in Models 2 and 3, as the group effect on absolute clock-checking in Model 2 was not significant, but the group effect on relative clock-checking in Model 3 was moderate (β = − 0.33).

#### Effects of time monitoring on TBPM performance

Noteworthy is that absolute and relative clock-checking both predicted TBPM performance (*β* = 0.54 and *β* = 0.50, respectively) in Model 1. To test the difference between the coefficients of two clock-checking measures on TPBM, we fit an alternative model (1b), in which the coefficients were constrained to be equal. There was no difference in the fit of Models 1 and 1b (Δχ^2^(1) = 1.487, *p* = 0.223), thus, checking the time more frequently and strategically equally contributed to TBPM performance. Models 2 and 3 yielded similar effects, as TBPM performance was predicted by absolute clock-checking in Model 2 (*β* = 0.51) and by relative clock-checking in Model 3 (*β* = 0.46).

Clinical group independently accounted for 3.3% of TBPM performance and 10.7% of relative clock-checking. When investigated separately, absolute and relative clock-checking accounted for 23.8% and 22.1% of TBPM performance respectively. Together, all predictors in Model 1 explained 53.9% of TBPM performance.

## Discussion

The present study addresses two important gaps in children’s PM literature related to ADHD. First, we replicated key results of previous studies with a free-paced, more ecologically relevant task representing everyday scenarios in a stimulus-rich VR environment. As in most of the previous studies^21–24^^; cf.^^25^, children with ADHD displayed lower TBPM performance compared to their typically developing peers. Second, by disentangling the roles of frequency of time monitoring and the level of strategic time monitoring in a single analysis, our study sheds light on the behavioral mechanisms underlying differences in TBPM between children with and without ADHD. Even though the participants in both groups equally monitor time overall, children with ADHD showed lower TBPM performance because they tended to check the clock more frequently during less critical moments compared to their typically developing peers.

### Mechanisms underlying TBPM challenges in children with ADHD

In the present study, we found support for our hypothesis and replicated previous results showing that children with ADHD had more difficulty remembering to carry out a given intended task at a specific moment than typically developing children^22–24^. A previous study^35^ found that children with and without ADHD perform similarly in a repetition task, which only requires verbally repeating task instructions but not executing them. This suggests ADHD-related difficulties in TBPM stem from the prospective (i.e., time estimation abilities and strategic monitoring to detect *when* to perform the PM task^16^) rather than the retrospective component of the task (i.e., remembering *what* to do). Accordingly, the present results showed that group-related differences in strategic time monitoring fully accounted for the lower TBPM performance of children with ADHD compared to their typically developing peers. This aligns with our hypothesis that time monitoring mediates ADHD-related challenges in TBPM.

When looking in more detail into the specific role of the two aspects of time monitoring, we first replicated and extended results from Voigt et al.^50^ by showing that checking the time often and strategically were not only separately, but also concomitantly, associated with better TBPM performance in both typically developing children and children with ADHD. We also found support for our hypothesis and replicated previous findings showing that children with ADHD did not check the clock more often (absolute time monitoring) than typically developing children, which is contrary to what could be expected due to their impulsivity^22–24^. Extending results from Zinke et al.^24^, the present study instead showed that group-related differences in TBPM stemmed specifically from a less strategic time monitoring pattern in children with ADHD than in typically developing children, after controlling for the overall frequency of time monitoring.

Taken together, these findings support the suggestion made by Joly-Burra et al.^26^ that absolute and relative clock-checking assess different and complementary aspects of time monitoring. While children with ADHD did not differ significantly from their typically developing peers in total time monitoring (i.e., absolute time monitoring), they did differ in the strategic allocation of their monitoring behavior over time (i.e., relative time monitoring). This pattern suggests that absolute time monitoring reflects time monitoring behavior is not influenced by ADHD. Simultaneously, children with ADHD seem to be less effective in strategic time monitoring, which may stem from difficulties in task prioritization and proactive control (see ^51,52^) as well as time-estimation^23^. With respect to the cognitive processes underlying time monitoring in PM, Munaretto et al.^20^ proposed a two-stage model in which an initial phase of loose time monitoring is followed by a phase of more fine-grained monitoring as the PM deadline approaches. In line with the model, absolute clock-checking may reflect general engagement and alertness, but not necessarily strategic control. Clock-checks during early intervals may also serve as a calibration function for time estimation, which the absolute frequency of clock-checking may partially capture. In contrast, and consistent with the second stage of the model, relative clock-checking is thought to reflect anticipatory goal maintenance, temporal planning, and the intentional allocation of attention (see also^26–28^). These are key aspects of proactive control.

The results also align with the attentional-gate model of PM, in which a core assumption is that accurate time estimation depends on adequate attention allocation between the ongoing task and time estimation for the PM duration^53^. According to this model, pulses from an internal pacemaker accumulate in a temporal register when the “gate” is open, that is, when attention is directed toward time. The model also posits that one can voluntarily update the internal representation of elapsed time (i.e., recalibrate) by externally monitoring for the time, with increasing frequency as the target time approaches. This predicted strategic time monitoring pattern is also thought to rely on attentional processes and proactive control.

In the context of ADHD, research suggests that the internal pacemaker may be accelerated due to dopaminergic dysregulation, leading to a faster accumulation of pulses and thus to an overestimation of elapsed time^54–58^. This could explain why children with ADHD begin monitoring too early—because they subjectively feel closer to the target time than they are. However, due to attentional control difficulties, they may fail to adjust their behavior adaptively after that initial check, resulting in inefficient time-monitoring dynamics. We view this mechanism as complementary to our interpretation that reduced relative clock-checking reflects diminished proactive control. While the faster pacemaker may influence when children start monitoring, weaker goal maintenance likely limits their ability to continue doing so strategically.

Regarding executive function and its three core functions as postulated by Miyake et al.^59^, Zuber et al.^60^ found that TBPM is associated with updating, but not with inhibition or shifting, in typically developing school-aged children. Nevertheless, given that challenges in inhibition are well-documented in ADHD (e.g.,^61^), inhibition may still contribute to attentional control difficulties, which may explain the current findings.

Given that attention allocation is a mechanism altered in ADHD, it is not surprising that children with ADHD are more challenged with accurate estimation of the PM interval duration, and thus also struggle to voluntarily deploy their time monitoring efforts strategically over time. One could hypothesize that children with ADHD struggle to harness their attentional resources to purposefully use their previous external time monitoring experience(s) to inform them on when it is more strategic to check the time next, and/or shield this latter intention from distractions.

By embedding PM tasks into real-life scenarios that recruit multiple cognitive processes (e.g., retrospective memory, executive functioning, planning, and monitoring), EPELI appears to be a promising tool to dissect and partial out the specific mechanisms through which ADHD-related challenges occur in complex everyday tasks. Furthermore, because EPELI is a VR task, it also measures a wide variety of behavioral measures that are usually not accessible in traditional PM paradigms. For instance, eye tracking data could provide further insight into children’s attention allocation strategies and help clarify how ADHD specifically influences attention directed toward time monitoring and time-estimation.

### Towards ecologically relevant clinical assessment of TBPM

Recent developments in VR technology, especially head-mounted displays, have stirred the interest to study PM in ecologically relevant conditions and thus increase the applicability of the results to everyday life^62–64^. In adults, both non-immersive VR implemented using traditional flat screen displays^65–67^ and immersive VR utilizing head-mounted displays^68,69^ have been successfully used to study PM. To the best of our knowledge, the current study is the first to harness the potential of VR to study PM in school-aged children with ADHD. As a method that allows precise measurement of continuous behavior in realistic yet well-controlled situations that emulate real-life conditions, VR offers promising new opportunities for PM research. For example, VR-based PM paradigms allow testing the generalizability of previously obtained findings and testing the theories in naturalistic setups (see Rummel and Kvavilashvili^70^). Furthermore, the higher verisimilitude of function-led VR scenarios to actual daily life situations, when compared to verisimilitude of simplified PM paradigms, may also support the development of related behavioral interventions^63^.

In the present study, we demonstrated that differences in TBPM performance with and without ADHD, observed earlier in traditional PM paradigms^22–24^, can also be captured using a naturalistic task. Although we cannot conclude that EPELI measures the exact same cognitive constructs as more traditional TBPM paradigms, the present findings indicate that TBPM-related difficulties in children with ADHD are robust enough to be detected with many types of tasks, including those that resemble real-world situations. The current findings are also in line with our previous study^35^ reporting that EPELI measures are associated with performance in other PM tasks, such as the Cruiser task^71^ and Heidelberger Exekutivfunktionsdiagnostikum Task^72^.

As described in the introduction, successful PM functioning plays a key role in everyday life of children (see also^8,73^). While traditional PM tasks with simplified stimuli are often considered advantageous in teasing apart different mechanisms involved in a broader cognitive function such as PM, we showed that by employing specific metrics, also ecologically relevant tasks can be used to pinpoint differences in how children with and without ADHD perform TBPM tasks and monitor time. Bridging the gap between the assessment context and situations where problems manifest could help in developing support for healthcare purposes. Efficient ways to monitor time could, for instance, be trained using a VR-based rehabilitation tool (e.g., one adapted from EPELI) where the difficulty of the TBPM tasks is adaptive depending on the task performance and successful performance is rewarded (see Romero-Ayuso et al.^74^ and Corrigan et al.^75^ for VR rehabilitation in ADHD). TBPM could be an important target for future VR rehabilitation studies. For instance, Geurten et al.^76^ showed that training metamemory may help children to learn strategic monitoring and improve their TBPM performance, while untrained controls relied more heavily on their executive functions. In other words, training meta-memory could help with the challenges resulting compromised executive functions and allow children to manage time-based tasks despite their ADHD symptoms.

### Limitations and outlook

Certain restrictions should be kept in mind when evaluating the results of the current study. Foremost, the delay between the TBPM instruction and target time in EPELI is rather short, varying from roughly half a minute to one minute. As real-life TBPM events are often, but not always, longer, this may be considered a limitation to the generalizability of the results. On the other hand, observing that children with ADHD show difficulties in strategic time monitoring and TBPM performance compared to their typically developing peers within such a short time span may indicate the robustness of the phenomenon as TBPM tasks tend to become more difficult when the target intervals become longer. Further research with longer intervals, for instance, using real-world experiments, should nevertheless be conducted to test if the results hold in various of everyday life situations. Testing PM over longer intervals may also allow for clearer differentiation between difficulties in estimating the passage of time and challenges in remembering when to initiate a given task.

Another limitation is that no subjective evaluation of children’s real-world PM skills was obtained from parents, teachers or children themselves. Comparing the TBPM performance to such measure would confirm the ecological validity of the results in terms of veridicality (see ^77^). Furthermore, this would have allowed us to study if children with ADHD have poorer metamemory or insight on their ability to perform tasks that need to be done at a specific time in the future^76^. In addition to attentional differences, children with ADHD may experience challenges related to meta-cognition and other domains of memory that could potentially be addressed in future studies^78^.

PM undergoes substantial improvements during school age, becoming more robust as children gain greater cognitive control and self-regulatory capacities (e.g.,^8,29,71,73,79^). Given that children with ADHD may differ from their typically developing peers in the pace of these processes, future research should aim to replicate the findings of this study with both younger (i.e., under nine years) and older children (i.e., over 12 years). This would help clarify whether children with ADHD exhibit different developmental trajectories of TBPM, particularly in terms of strategic monitoring. Wang et al.^29^ found that between the ages of 7 and 11 years, typically developing children become increasingly skilled at using their attentional resources for time monitoring. This developmental progression may be delayed in children with ADHD, or ADHD may be associated with persistent difficulties in strategic time monitoring into adolescence.

Given that reward plays a significant role in ADHD^80^, future studies should also focus on clarifying the role of motivational factors in modulating differences in PM performance between children with and without ADHD (see, e.g.,^81,82^). In an earlier study comprising half of the current data, children with and without ADHD reported similar levels of enthusiasm and effort when performing EPELI^36^. Therefore, group differences in PM performance might be magnified in less motivating tasks. Besides attractiveness, the distractibility of objects in the virtual environment could be considered more closely. We have previously shown that children with ADHD display lower task efficiency in EPELI by performing more actions irrelevant to the given tasks than typically developing controls^35,38^. However, the interactions between accuracy and timing of actions and different stimulus features modulating TBPM performance should be examined in a separate study, ideally with larger sample and specific experimental design tailored for examining attractiveness and distractibility of the stimuli. Furthermore, the roles of different ADHD symptoms, namely inattention, hyperactivity, and impulsivity, on TBPM performance could be examined using larger datasets with greater statistical power.

Cognitive challenges and lower socioeconomic status, including lower income, are commonly associated with ADHD (e.g.,^83–85^). This pattern is reflected in our data, with the ADHD group showing lower performance on reasoning tasks and reporting lower parental income. Given prior concerns that matching groups or statistically controlling for variables inherently linked to a clinical condition can lead to anomalous or misleading interpretations of neurocognitive function^86^, we chose not to apply such adjustments in the present study, which focuses on TBPM and time monitoring. However, to assess the robustness of our findings, we also conducted analyses using propensity score matching based on gender, age, verbal reasoning, non-verbal reasoning, and family income. The overall pattern of results remained consistent. Future research could explore the potential mediating role of reasoning abilities in the relationship between ADHD and PM, ideally incorporating both time-based and event-based PM tasks.

Finally, as PM is associated with executive function in childhood^8,60^ and throughout the lifespan^15^, further studies should be conducted to probe the possible mediating roles of specific executive function components (e.g., working memory, inhibition and set shifting) to the association between ADHD and PM (see ^8,87^).

## Conclusion

Our novel findings demonstrate that specific mechanisms underlying differences in TBPM performance among children with and without ADHD can be studied in a naturalistic VR environment that simulates everyday home situations. The results of Seesjärvi et al.^35^ and the current study suggest that these challenges might not be explained by motivation but by higher-level cognitive processes and behavioral mechanisms required to monitor time at relevant moments. As ADHD is common^12^ and can, when untreated, have severe consequences in different life domains^88,89^, identifying specific mechanisms explaining PM difficulties that constitute a large proportion of everyday memory problems could have a considerable societal impact. Our results indicate that previous findings obtained with traditional methods generalize to an ecologically relevant task, providing further cross-validation that these methods are well-suited for identifying TBPM deficits associated with ADHD. Overall, the findings of the present study could inform clinical assessments of cognitive functioning in childhood ADHD, guide the development of related interventions, and serve as a foundation for future research directions.

## Supplementary Information

Supplementary Information.

## Data Availability

In compliance with the research permission by the Ethics Committee of the Helsinki University Hospital, supporting data for this study is not available due to patient confidentiality restrictions. Requests to access the datasets should be directed to erik.seesjarvi@helsinki.fi.
