# Supplementary material for: A naturalistic virtual reality task reveals difficulties in time-based prospective memory and strategic time-monitoring in children with ADHD
Source: Sci Rep. 2025 Jul 9;15:24722. doi: 10.1038/s41598-025-08944-w (PMC12241525; doi:10.1038/s41598-025-08944-w)
Supplement: Supplementary file 1 — Supplementary Information. [file 41598_2025_8944_MOESM1_ESM.docx]

# Supplementary material for “*A naturalistic virtual reality task reveals difficulties in time-based prospective memory and strategic time-monitoring in children with ADHD”*

|  |  | Group | |  |  |
| --- | --- | --- | --- | --- | --- |
| Variable |  | ADHD  (n = 71) | Control  (n = 82) | Test statistics | *p* |
| Age (Mean (SD)) |  | 10.4 (1.1) | 10.8 (1.0) | *t*(151) = -2.536 | .01* |
| Gender | boy | 58 | 49 | Fisher's Exact Test | .004** |
|  | girl | 13 | 33 |  |  |
| Parental income before tax (%) | less than 1,500 €/m | 2 (2.8) | 0 (0.0) | W = 1934.5^a^ | < .001*** |
|  | 1,500–2,200 €/m | 4 (5.6) | 2 (2.8) |  |  |
|  | 2,200–3,000 €/m | 27 (38.0) | 10 (14.1) |  |  |
|  | 3,000–4,000 €/m | 15 (22.5) | 20 (28.2) |  |  |
|  | over 4 000 €/m | 22 (31.0) | 39 (54.9) |  |  |
| Verbal reasoning (Mean (SD)) |  | 10.4 (2.7) | 11.5 (2.5) | *t*(151) = -2.734 | .007** |
| Non-verbal reasoning (Mean (SD)) | | 9.5 (3.1) | 10.7 (3.3) | *t*(151) = -2.289 | .023* |
| ADHD-RS (Mean (SD)) |  | 32.0 (8.9) | 6.8 (6.2) | *t*(151) = 20.579 | < .001*** |

Supplementary Table 1. Background variables for the sample before propensity matching. SD = standard deviation. Verbal reasoning = WISC-IV Similarities standard score. Non-verbal reasoning = WISC-IV Matrix reasoning standard score. ADHD-RS = ADHD Rating Scale-IV. ^a^ = Wilcoxon rank sum test with continuity correction. * = p < .05, ** = p < .01, *** = p < .001.

## Supplementary Methods

### An example scenario from EPELI

The scenario is titled "Leaving for football (soccer) practice." It begins in the child’s room (see Supplementary Figure 1) with the encoding phase, during which a cartoon dragon, Laura the Dragon, appears and gives the following instructions (with the TBPM task highlighted in bold):

*Now you're getting ready to go to football practice. Get your shoes and ball from next to your bed and place them by the door, be ready to take them with you. Make your bed — you forgot to do it in the morning. Remember to answer the phone when it rings, because your friend will call you when they're outside.* ***When it's 2 o'clock, turn on the oven.*** *If you have time while waiting, you can watch a video. Turn off the lights when you leave the house.*

In this scenario, the encoding phase lasts 42 seconds, after which the execution phase begins. The child is required to complete six different tasks:

1. Get their shoes and ball and place them by the door.
2. Make their bed.
3. Answer the phone.
4. Turn on the oven (at 2 p.m.) — TBPM task.
5. Watch a video (by turning on the TV).
6. Turn off the lights.

Each successfully completed task is awarded one point. Task #4 (turning on the oven at 2 p.m.) is the TBPM task, while the other tasks are considered ongoing tasks. The scenario ends either when all tasks are completed or after a maximum of 90 seconds.


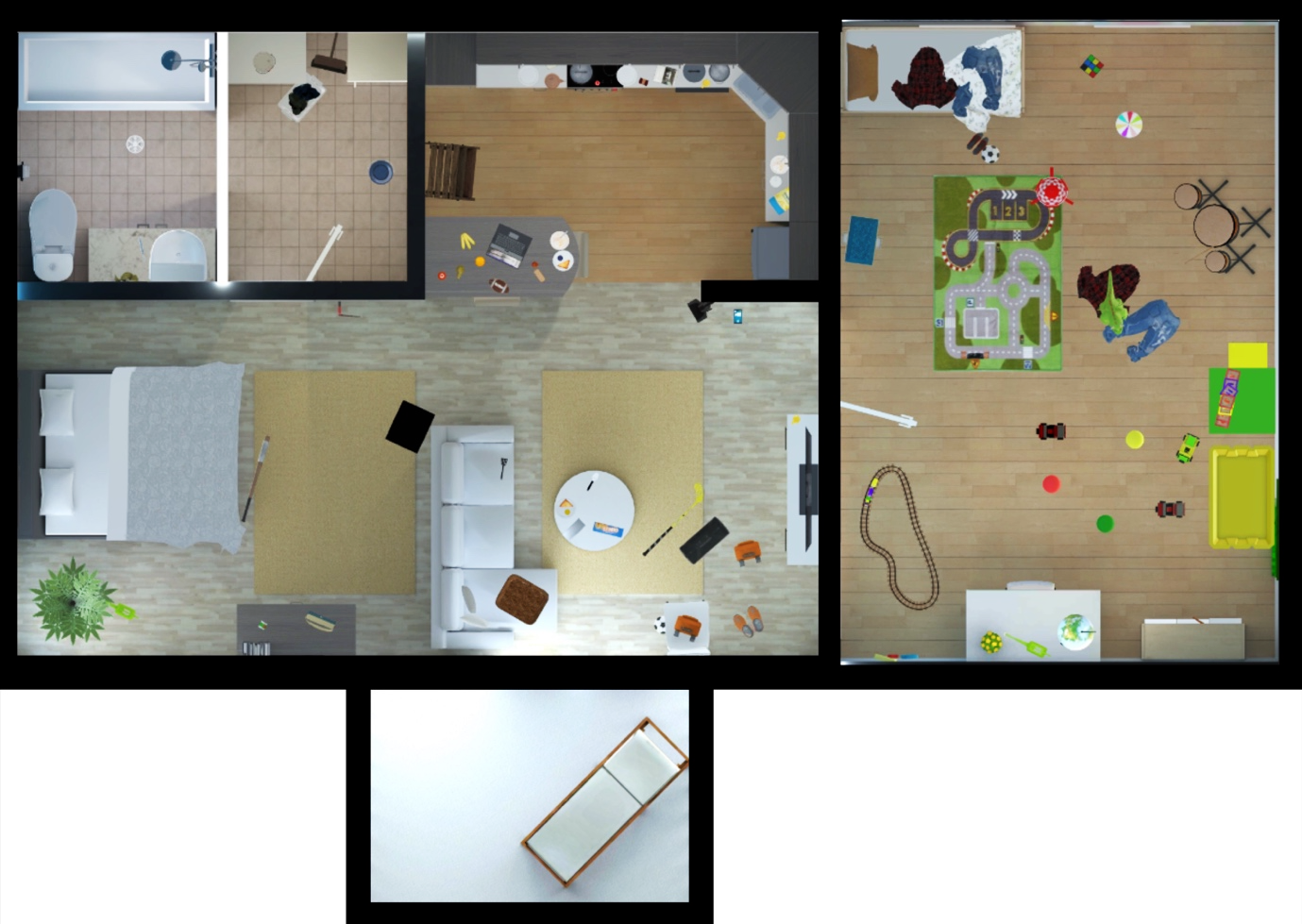
Supplementary Figure 1. Floor plan of the apartment in EPELI (**not** shown to participants). In the upper left corner, a bathroom and utility room are visible, and below them, an open adult bedroom. In the center, there is an open kitchen and living room. On the right side of the figure, a children's room can be seen. The balcony, shown at the bottom of the figure, was not accessible but was visible through the windows.
